# β-arrestin-1 contributes to brown fat function and directly interacts with PPARα and PPARγ

**DOI:** 10.1038/srep26999

**Published:** 2016-06-15

**Authors:** Congcong Wang, Xianglu Zeng, Zhaocai Zhou, Jian Zhao, Gang Pei

**Affiliations:** 1 State Key Laboratory of Cell Biology, Institute of Biochemistry and Cell Biology, Shanghai Institutes for Biological Sciences, Chinese Academy of Sciences, 320 Yueyang Road, Shanghai 200031, China; 2 Graduate School, University of Chinese Academy of Sciences, Chinese Academy of Sciences, 320 Yueyang Road, Shanghai 200031, China; 3 Shanghai Key Laboratory of Signaling and Disease Research, Laboratory of Receptor-based Bio-medicine, School of Life Sciences and Technology, Tongji University, Shanghai 200092, China; 4 Translational Medical Center for Stem Cell Therapy, Shanghai East Hospital, School of Medicine, Tongji University, Shanghai 200120, China; 5 School of Life Science and Technology and the Collaborative Innovation Center for Brain Science, Tongji University, Shanghai 200092, China

## Abstract

The peroxisome proliferator-activated receptor (PPAR) family plays central roles in brown adipose tissue (BAT) adipogenesis and contributes to body temperature maintenance. The transcriptional activity of PPAR family has been shown to be tightly controlled by cellular signal networks. β-arrestins function as major secondary messengers of G protein-coupled receptors (GPCR) signaling by functional interactions with diverse proteins. Here, we report that β-arrestin-1 knock-out mice show enhanced cold tolerance. We found that β-arrestin-1 directly interacts with PPARα and PPARγ through a LXXXLXXXL motif, while D371 in PPARα and L311/N312/D380 in PPARγ are required for their interactions with β-arrestin-1. Further mechanistic studies showed that β-arrestin-1 promotes PPARα- but represses PPARγ-mediated transcriptional activities, providing potential regulatory pathway for BAT function.

Brown adipose tissue (BAT) produces heat through the process of non-shivering thermogenesis, which contributes to the maintenance of body temperature in response to cold challenge^1^,^2^. Cold exposure activates the sympathetic nervous system, resulting in the stimulation of β-adrenergic receptors in brown adipocytes, leading to the activation of oxidative phosphorylation and thermogenic activity. This process is facilitated by the enhanced expression of mitochondrial uncoupling protein-1 (UCP1), which uncouples fatty acid oxidation from ATP production, and thereby releases chemical energy as heat^3^,^4^,^5^.

The PPAR family plays central roles in brown fat adipogenesis and body temperature control. As a major activator of mitochondrial and peroxisomal fatty acid oxidation^6^, PPARα is involved in the activation of UCP1 and BAT-mediated body temperature control^7^,^8^,^9^,^10^. PPARα promotes brown fat adipogenesis in cooperation with PGC1α, SRC-1, and PRDM16^11^,^12^. On the other hand, in cooperation with CCAAT/enhancer-binding protein family members (C/EBPs), PPARγ functions as a master regulator of adipocyte differentiation^13^ and promotes the transcription of adipogenic genes^14^,^15^,^16^,^17^. The transcriptional activity of PPAR family has been shown to be controlled by recruiting diverse cofactors^18^.

β-arrestins mediate desensitization and endocytosis of G protein-coupled receptors (GPCR) and have been considered as GPCR signal terminators^19^. Further studies have demonstrated that β-arrestins regulate diverse signaling pathways by acting as scaffolds in numerous protein complexes^20^. Our previous studies have shown that β-arrestin-1 interacts with PPARγ and represses PPARγ-mediated adipogenesis and inflammatory responses in white adipose tissue (WAT)^21^,^22^. In this study, we report that β-arrestin-1 directly interacts with PPARα and PPARγ. We identified that the LXXXLXXXL motif in β-arrestin-1 is required for the interaction with PPARα and PPARγ. In addition, we found that D371 in PPARα and L311/N312/D380 in PPARγ are required for their interactions with β-arrestin-1. Our mechanistic studies showed that β-arrestin-1 enhances PPARα-mediated transcriptional activity but represses PPARγ-dependent gene expression, and thus contributes to BAT function.

## Results

### Arrb1-KO mice show enhanced cold tolerance and increased thermogenic gene expression

β-arrestin-1 knock-out (Arrb1-KO) mice were generated by deletion of exons 2 and 3 in *Arrb1*, which were flanked by two loxP sites. The frt-Neo-frt cassette was used as a positive selection marker to get the embryonic stem cells, which were then injected into the C57BL/6 blastocysts. The resulting embryos were transferred to pseudopregnant mice to generate the chimeric mice. We mated male founders and female *Cre*^*Tg/Tg*^ mice to obtain *Arrb1*^*Flox*/+^;*Cre*^*Tg*/+^ mice, which were then crossed with C57BL/6 mice to remove the *Cre* gene and back-crossed 10 times to the C57BL/6 background. All mice were genotyped by PCR analysis of genomic DNA. The resulting Arrb1-KO mice showed no detectable β-arrestin-1 left (Supplementary Fig. 1).

**Figure 1:**
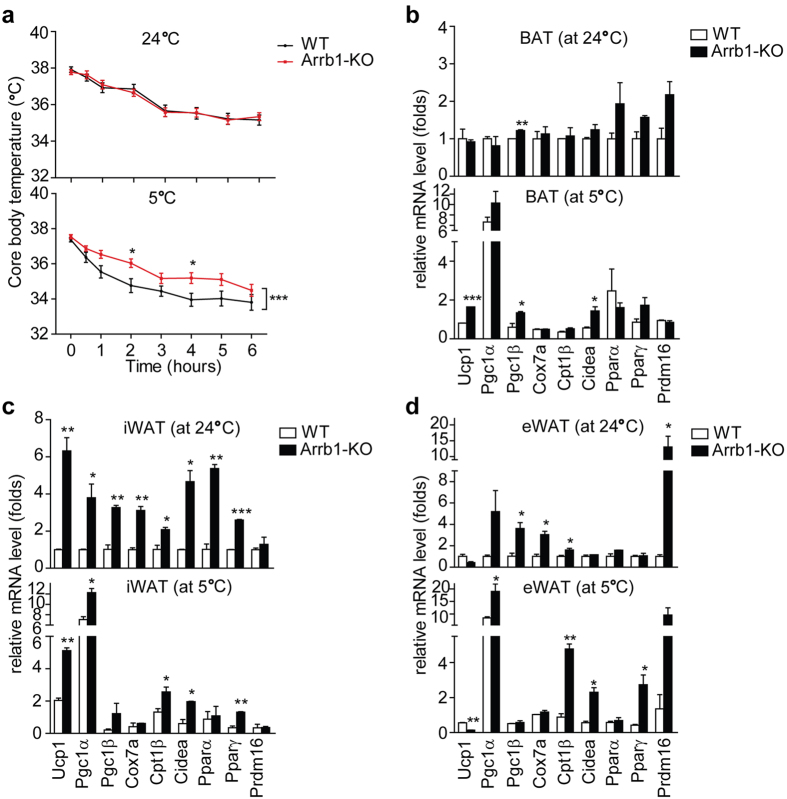
Arrb1-KO mice show enhanced cold tolerance and increased thermogenic gene expression. (**a**) Rectal temperatures of wild-type (n = 13) and Arrb1-KO (n = 13) mice during exposure to 24 °C or 5 °C for 6 hours. Data are shown as means ± S.E.M. *p < 0.05 and ***p < 0.001 *versus* the wild-type group, as determined by two-way ANOVA followed by Bonferroni post hoc tests. (**b–d**) Real-time quantitative PCR analysis of thermogenic gene expression in BAT, iWAT, and eWAT respectively. Tissues were harvested from the wild-type and Arrb1-KO mice after exposure to 24 °C or 5 °C for 6 hours. Data are presented as means ± S.E.M. *p < 0.05, **P < 0.01, and ***P < 0.001 *versus* the wild-type group, as determined by unpaired two-tailed Student’s *t* test.

We used Arrb1-KO mice to investigate the role of β-arrestin-1 in BAT thermogenesis. We found that at room temperature (24 °C), when nonshivering thermogenesis is not required, there were no differences in core body temperature between the wild-type and Arrb1-KO mice. However, when we exposed the mice to an ambient temperature of 5 °C, the body temperatures of Arrb1-KO mice were about 1 °C higher compared with that of the wild-type mice after 2 hours or 4 hours of cold exposure, suggesting an enhanced thermogenic capacity after cold challenge in Arrb1-KO mice (Fig. 1a). We further monitored the expression of key thermogenic genes and fatty acid oxidation genes, including *Ucp1*, *Cidea*, *Pgc1*α, *Pgc1*β, *Cox7a*, and *Cpt1*β. In BAT, the expression of *Ucp1*, *Cidea*, and *Pgc1β* was considerable increased in Arrb1-KO mice after cold exposure compared with the wild-type mice (Fig. 1b). In subcutaneous inguinal adipose tissue (iWAT), we observed a noticeable increase in *Ucp1*, *Pgc1*α, *Cidea*, and *Cpt1β* mRNA levels in Arrb1-KO mice compared with that of their wild-type littermates (Fig. 1c). In epididymal adipose tissue (eWAT), the mRNA levels of *Pgc1*α, *Cidea*, and *Cpt1β* were remarkably increased in Arrb1-KO mice after cold exposure compared with that of the wild-type mice (Fig. 1d). Taken together, these results showed that deficiency of β-arrestin-1 enhanced cold tolerance and increased thermogenic gene expression *in vivo*.

### β-arrestin-1 directly interacts with PPARα and PPARγ and interferes with the formation of PPARs/RXRα heterodimers

**Figure 2:**
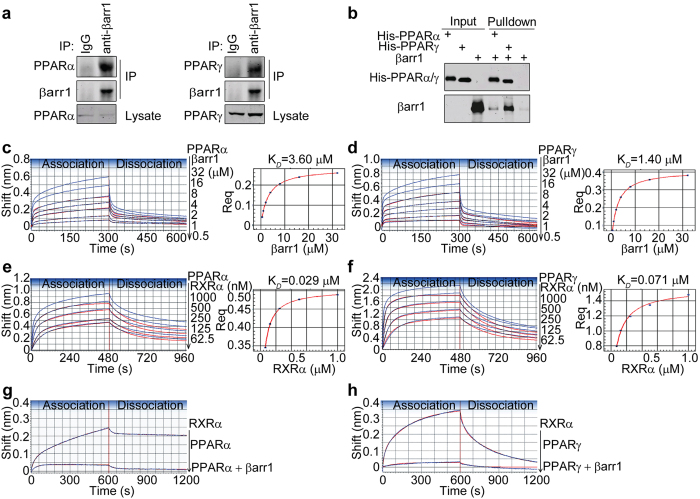
β-arrestin-1 directly interacts with PPARα and PPARγ and interferes with the formation of PPARs/RXRα heterodimers. (**a**) Interactions between endogenous β-arrestin-1 and PPARα/γ. Lysates from HepG2 cells were subjected to immunoprecipitation (IP) using mouse anti-β-arrestin-1 antibody. The immunopurified complexes were shown on immunoblots. (**b**) Interactions of β-arrestin-1 with PPARα/γ-LBD were analyzed in an *in vitro* pulldown assay. Purified β-arrestin-1 was incubated with his-tagged PPARα/γ-LBD immobilized on Ni-NTA agarose beads. The purified protein complexes were detected on Western blot with mouse anti-His and anti-β-arrestin-1 antibodies. The immunoblots in (**a,b**) were run under the same experimental conditions. Cropped blots were shown in (**a,b**) and the full-length blots were presented in Supplementary Figure 11. (**c,d**) Biolayer interferometry analysis of the interactions between β-arrestin-1 and PPARα/γ-LBD respectively using the ForteBio Octet Red instrument. The steady state analysis of the binding curves and the equilibrium dissociation constants K_*D*_ were shown on the right. Streptavidin biosensors (SAs) immobilized with 50 μg/ml biotinylated PPARα-LBD or PPARγ-LBD were incubated in wells containing different concentrations of purified β-arrestin-1 at 25 °C. (**e,f**) The kinetic analysis of RXRα-LBD binding to PPARα-LBD and PPARγ-LBD, respectively. The steady state analysis and K_*D*_ of the binding curves were shown on the right. Purified 50 μg/ml RXRα-LBD was loaded on SAs and incubated with serial dilutions of purified PPARα/γ-LBD solution at 25 °C. (**g,h**) The kinetics of RXRα-LBD binding to PPARα-LBD and PPARγ-LBD in the presence of β-arrestin-1. 200 nM PPARα-LBD and PPARγ-LBD were incubated with RXRα-LBD-loaded SAs in the absence or presence of β-arrestin-1 at 25 °C. The 2:1 heterogeneous ligand (HL) model was used to fit all the association/dissociation steps. The experimental data are represented by blue lines and the curve fitting data are indicated by red lines.

β-arrestins have been reported to function as signal adaptors by interactions with diverse proteins^20^. Studies have shown that PPARα and PPARγ play critical roles in BAT thermogenesis^7^,^17^,^23^. We hypothesized that β-arrestin-1 might interact with PPARα and PPARγ to regulate BAT-mediated body temperature control. We first examined the endogenous interactions between β-arrestin-1 and PPARα/γ by immunoprecipitation. PPARα and PPARγ were detected in the immunopurified β-arrestin-1 complex (Fig. 2a). To further confirm the direct interactions of β-arrestin-1 with PPARα/γ, we purified β-arrestin-1 and PPARα/γ-ligand binding domain (LBD) from *E. coli*. In an *in vitro* pulldown assay, we observed that β-arrestin-1 bound to his-tagged PPARα-LBD and PPARγ-LBD (Fig. 2b).

We then used biolayer interferometry to conduct a kinetic analysis of the interactions between β-arrestin-1 and PPARα/γ-LBD. Purified 50 μg/ml PPARα-LBD and PPARγ-LBD were biotinylated and immobilized on streptavidin biosensors (SAs). The SAs were then incubated in wells containing different concentrations of purified β-arrestin-1 (0.5–32 μM). Representative data for association/dissociation phases of the curves were demonstrated in Fig. 2c,d (the experimental data are represented by blue lines and the curve fitting data are indicated by red lines). We observed temporary and quick initial association/dissociation steps, followed by much longer and slower steps in the binding curves of β-arrestin-1 with PPARα/γ-LBD. Similar maximum responses in the binding curves of β-arrestin-1 with PPARα-LBD or PPARγ-LBD were observed. Global fitting of the experimental data generated a best fit with the 2:1 heterogeneous ligand (HL) model, indicating the existence of two ligand binding sites. Therefore, two values of association/dissociation rate constants (k_*on*_ and k_*off*_) and affinities (K_*D*_) were presented in Tables 1 and 2. The ratio of these two kinetic interactions in the total binding has been determined by the two calculated Rmax parameters (Rmax1 and Rmax2), which reflect the proportion of each interaction contributing to the overall signal at saturation. In contrast, the fitting curves generated from the 1:1 interaction, mass transport, 1:2 bivalent analyte (BA) model did not match the experimental data well, especially at the initiation phase of the association/dissociation steps. The residual plots derived from the 2:1 HL fitting model showed small residuals (Supplementary Fig. 4). The R^2^ values were above 0.97, and the χ^2^ values were below 0.90 for all fits. Additionally, steady-state analysis was demonstrated in Fig. 2c,d (right panels), in which the estimated response at equilibrium for each analyte concentration rather than the k_*on*_ and k_*off*_ values was used. The single steady-state equilibrium dissociation constants K_*D*_ and the Rmax which represents the saturating binding level were presented in Tables 1 and 2.

We further examined the interactions of PPARα/γ-LBD with 9-cis-retinoic acid receptor α-ligand binding domain (RXRα-LBD) in the absence or presence of β-arrestin-1. We found that β-arrestin-1 showed no detectable interaction with RXRα-LBD (Supplementary Fig. 5a). As shown in Fig. 2e–h, the maximum responses of the interactions between PPARα/γ-LBD and RXRα-LBD were reduced after pre-incubation of PPARα/γ-LBD with β-arrestin-1. We also assessed the interactions between PPARα/γ-LBD and β-arrestin-1 with or without RXRα-LBD. Reduced maximum responses of the interactions between PPARα/γ-LBD and β-arrestin-1 were observed in the presence of increased amount of RXRα-LBD. As shown in Supplementary Fig. 5b,c, RXRα-LBD competed with β-arrestin-1 for the binding of PPARα-LBD (IC_50_ = 0.88 μM) or PPARγ-LBD (IC_50_ = 0.85 μM). These results suggest that β-arrestin-1 directly interacts with PPARα-LBD and PPARγ-LBD. Furthermore, β-arrestin-1 and RXRα-LBD compete for the interaction with PPARα-LBD or PPARγ-LBD.

### β-arrestin-1 directly interacts with PPARα and PPARγ via a LXXXLXXXL motif and regulates the transcriptional activities of PPARs

**Figure 3:**
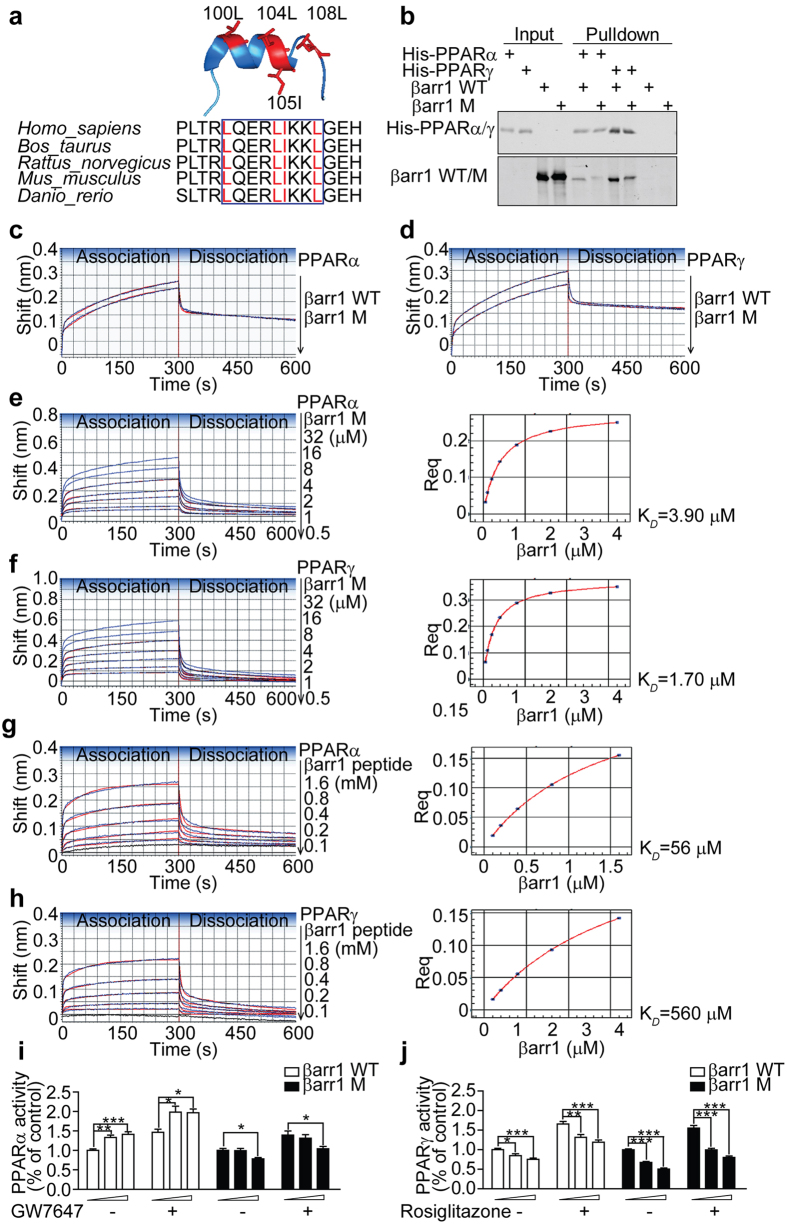
β-arrestin-1 directly interacts with PPARα and PPARγ via a LXXXLXXXL motif and regulates the transcriptional activities of PPARs. (**a**) Sequence alignment (down) and secondary structure (up) of the LXXXLXXXL motif in β-arrestin-1. (**b**) Interactions of βarr1M with PPARα/γ-LBD using an *in vitro* pulldown assay. Purified His-tagged PPARα/γ-LBD were incubated with the wild-type β-arrestin-1 or βarr1M and subjected to anti-His antibody. The immunoblots in (**b**) were run under the same experimental conditions. Cropped blots were shown in (**b**) and the full-length blots were presented in Supplementary Figure 11. (**c,d**) Biolayer interferometry analysis of the interactions between βarr1M and PPARα/γ-LBD. The SAs loaded with 50 μg/ml PPARα/γ-LBD were incubated with 2 μM purified wild-type β-arrestin-1 or βarr1M at 25 °C. (**e,f**) The kinetic analysis of βarr1M binding to PPARα/γ-LBD. The steady state analysis and K_*D*_ of the binding curves were shown on the right. The SAs immobilized with 50 μg/ml PPARα/γ-LBD were incubated with different concentrations of purified βarr1M at 25 °C. (**g,h**) The kinetics of interactions between β-arrestin-1 peptide and PPARα/γ-LBD. The steady state analysis and the single dissociation constants K_*D*_ were shown on the right. 50 μg/ml biotinylated PPARα-LBD and PPARγ-LBD were loaded on SAs and incubated with an irrelevant control peptide (black lines) or a serial dilution of β-arrestin-1 peptide (blue lines) at 25 °C. The 2:1 HL model was used to fit all the association/dissociation steps. The experimental data are represented by blue lines and the curve fitting data are indicated by red lines. (**i,j**) PPRE-driven luciferase activity in HEK-293T cells co-transfected with PPARα (**i**) /PPARγ (**j**) and the wild-type β-arrestin-1 or βarr1M, in the absence or presence of agonists. Data were normalized to the groups which were co-transfected with PPARα/γ and β-gal in the absence of agonists (mean ± S.E.M from three independent experiments). *p < 0.05, **P < 0.01, and ***P < 0.001, as determined by one-way ANOVA.

The transcriptional activities of PPARs are mediated through their binding with diverse cofactors, which carry a conserved α-helix motif, typically with sequences of LXXXI/LXXXI/L or LXXLL^24^,^25^. To explore the potential binding motif in β-arrestin-1 with PPARα and PPARγ, we examined the tertiary structure of β-arrestin-1, which comprises of two domains of antiparallel β-sheets and one short α-helix (residues 98–108)^26^. The alignment of β-arrestin-1 families showed a conserved sequence of LQERLIIKKL in the α-helix, where the hydrophobic residues Leu100, Leu104, and Leu108 are aligned on the same face of the helix (Fig. 3a). We hypothesized that this motif in β-arrestin-1 might play a role in the interactions with PPARα and PPARγ. To test this, we generated a β-arrestin-1 double mutant with L100A and L104A (βarr1M). First, we evaluated the interactions of wild-type β-arrestin-1 and βarr1M with Gαs, which was reported to bind to Leu33 in β-arrestin-1^27^. As shown in Supplementary Fig. 6, βarr1M showed a similar binding profile with Gαs in comparison to the wild-type β-arrestin-1, suggesting a proper folding of βarr1M. Then we performed an *in vitro* pulldown assay. We found that compared with the wild-type β-arrestin-1, βarr1M showed reduced interactions with both his-tagged PPARα-LBD and PPARγ-LBD (Fig. 3b).

We also evaluated the kinetic properties of interactions between PPARα/γ-LBD and βarr1M. As shown in Fig. 3c–f, in comparison with the wild-type β-arrestin-1, βarr1M interacted with PPARα-LBD or PPARγ-LBD with about 20% impaired maximum responses (with PPARα-LBD, βarr1M vs. βarr1: 0.46 nm vs. 0.59 nm; with PPARγ-LBD, βarr1M vs. βarr1: 0.59 nm vs. 0.77 nm). In addition, βarr1M showed a slightly decreased binding affinity with PPARα-LBD (K_*D*_ of βarr1M vs. βarr1: 3.9 μM vs. 3.6 μM) or PPARγ-LBD (K_*D*_ of βarr1M vs. βarr1: 1.7 μM vs. 1.4 μM) compared to the wild-type β-arrestin-1. These results indicate that the residues Leu100 and Leu104 in LXXXLXXXL motif of β-arrestin-1 are required for the interactions with PPARα/γ-LBD.

To further test whether direct interactions between the LXXXLXXXL motif in β-arrestin-1 and PPARα/γ-LBD exist, we performed the biolayer interferometry assay using immobilized PPARα/γ-LBD and synthetic β-arrestin-1 peptides harboring the LQERLIKKL sequence. As shown in Fig. 3g,h, we observed that the synthetic β-arrestin-1 peptide bound to both PPARα-LBD and PPARγ-LBD in dose-dependent manners. In contrast, an irrelevant control peptide showed no detectable interaction with PPARα/γ-LBD. The binding profiles of β-arrestin-1 peptide with PPARα/γ-LBD were comparable to that of β-arrestin-1. However, reduced maximum responses in binding curves of β-arrestin-1 peptide with PPARα/γ-LBD were observed (with PPARα-LBD, βarr1 peptide vs. βarr1: 0.27 nm vs. 0.59 nm; with PPARγ-LBD, βarr1 peptide vs. βarr1: 0.22 nm vs. 0.77 nm), which might due to the relatively low molecular weight (~2 kDa) of β-arrestin-1 peptide. Meanwhile, the equilibrium dissociation constants K_*D*_ of β-arrestin-1 peptide with PPARα/γ-LBD were significant higher compared with β-arrestin-1 (Tables 1 and 2). We also found that β-arrestin-1 peptide, but not the control peptide with a similar molecular weight, interfered with the interactions between PPARα/γ-LBD and RXRα-LBD (Supplementary Fig. 7). We also performed the kinetic analysis of interactions between β-arrestin-1 peptide mutants and PPARα/γ-LBD. As shown in Supplementary Fig. 8, the β-arrestin-1 peptide double mutants with L100A and L104A (M1) or with L100E and L104E (M2) showed reduced interactions with PPARα/γ-LBD. Notably, β-arrestin-1 peptide M2 abolished the interaction with PPARα-LBD. All the kinetic parameters (k_*on*_ and k_*off*_) and affinities (K_*D*_) are presented in Tables 1 and 2. The residual plots derived from the the 2:1 HL fitting model were shown in Supplementary Fig. 10.

To further test whether the interactions of β-arrestin-1 with PPARα and PPARγ mediate the transcriptional activities of PPARs, we performed the reporter assay by measuring the luciferase activity under the control of a PPAR response element (PPRE). As shown in Fig. 3i, β-arrestin-1, but not βarr1M, increased PPARα activity in a dose-dependent manner in the presence or absence of a selective PPARα agonist GW7647. We also observed that β-arrestin-1 repressed the transcriptional activity of PPARγ with or without a PPARγ-specific agonist Rosiglitazone. However, we found βarr1M showed a similar transrepression activity of PPARγ compared to the wild-type β-arrestin-1 (Fig. 3j), possibly because of the presence of another interaction domain in β-arrestin-1 with PPARγ as shown in our previous report^21^. Taken together, these results indicate that β-arrestin-1 directly interacts with PPARα and PPARγ via a LXXXLXXXL motif and regulates the transcriptional activities of PPARα/γ.

### D371 in PPARα and L311/N312/D380 in PPARγ are required for their interactions with β-arrestin-1

**Figure 4:**
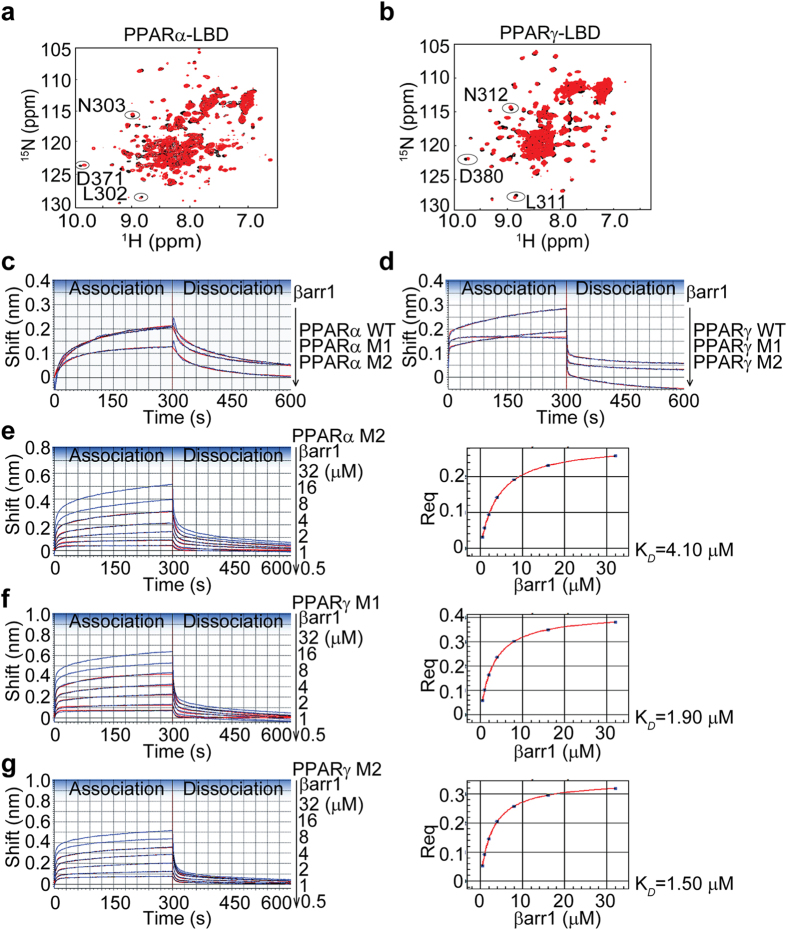
D371 in PPARα and L311/N312/D380 in PPARγ are required for their interactions with β-arrestin-1. (**a,b**) Overlays of the NMR spectra of apo-PPARα-LBD or PPARγ-LBD (black) and the proteins in complex with β-arrestin-1 peptide (red). ^15^N-labeled PPARα/γ-LBD were titrated with β-arrestin-1 peptide using a molar ratio up to 1:10. The 2D [1H, 15N]-HSQC spectra were recorded at 800 MHz. (**c**) Biolayer interferometry analysis of the interactions between PPARα-LBD L302G/N303G (PPARα M1) or D371A (PPARα M2) and β-arrestin-1. Purified 50 μg/ml wild-type PPARα-LBD, PPARα M1, and PPARα M2 were immobilized on SAs and incubated with 2 μM β-arrestin-1 at 25 °C. (**d**) Interactions between PPARγ-LBD L311G/N312G (PPARγ M1) or D380A (PPARγ M2) and β-arrestin-1 using the ForteBio Octet Red instrument. 50 μg/ml wild-type PPARγ-LBD, PPARγ M1, and PPARγ M2 were immobilized on SAs and incubated with 2 μM β-arrestin-1 at 25 °C. (**e–g**) The kinetic analysis of β-arrestin-1 binding to PPARα M2, PPARγ M1 and M2, respectively. The steady state analysis and K_*D*_ of the binding curves were shown on the right. 50 μg/ml PPARα M2, PPARγ M1 and PPARγ M2 were loaded on SAs and incubated with a serial dilution of purified β-arrestin-1 solution at 25 °C. The 2:1 HL model was used to fit the binding curves. The experimental data are represented by blue lines and the curve fitting data are indicated by red lines.

We further examined by nuclear magnetic resonance (NMR) spectroscopy the specific residues in PPARα and PPARγ that are critical for binding with β-arrestin-1. We carried out an NMR chemical shift perturbation assay in which a sample of ^15^N-labeled PPARα-LBD or PPARγ-LBD was titrated with β-arrestin-1 peptide up to a molar ratio of 1:10. Representative overlays of the 2D [1H, 15N]-HSQC spectra in the absence or presence of β-arrestin-1 peptide were shown in Fig. 4a,b. Several peaks altered considerably upon the titration of β-arrestin-1 peptide, indicating substantial conformational changes in PPARα-LBD and PPARγ-LBD. Using previous NMR data and assignments^28^, we extended the NMR chemical shift assignments of PPARγ-LBD for β-arrestin-1 peptide titration at amino acids L311, N312, and D380. Considering the sequence-structure homology and the similarity of chemical shift, we hypothesized amino acids L302, N303, and D371 of PPARα-LBD might be required for the binding with β-arrestin-1.

To confirm the potential binding sites in PPARα and PPARγ with β-arrestin-1, we generated the following four mutants: PPARα-LBD L302G/N303G (PPARα M1), PPARα-LBD D371A (PPARα M2), PPARγ-LBD L311G/N312G (PPARγ M1), and PPARγ-LBD D380A (PPARγ M2). Then we performed the biolayer interferometry assay. We found that PPARα M2, but not M1 showed a reduced interaction with β-arrestin-1 (Fig. 4c). In addition, both PPARγ M1 and M2 showed impaired interactions with β-arrestin-1 (Fig. 4d).

We then monitored the kinetics of β-arrestin-1 binding to PPARα M2, PPARγ M1, and PPARγ M2. As shown in Fig. 4e, compared with the wild-type PPARα, PPARα M2 showed a ~10% impaired maximum response with β-arrestin-1 (0.51 nm vs. 0.59 nm), while a comparable steady-state K_*D*_ was obtained. Likewise, compared with the wild-type PPARγ, PPARγ M1 and M2 showed slightly increased equilibrium dissociation constants K_*D*_ of interactions with β-arrestin-1 but reduced maximum responses (PPARγ Μ1 or Μ2 vs. PPARγ, 0.64 nm or 0.51 nm vs. 0.77 nm) (Fig. 4f,g). Consistent with these results, reduced maximum responses of βarr1M binding to PPARγ M1 and M2 were also observed (Supplementary Fig. 9). All the association/dissociation rate constants are presented in Tables 1 and 2. The residual plots derived from the 2:1 HL fitting model were shown in Supplementary Fig. 10. These results indicate that D371 in PPARα and L311/N312/D380 in PPARγ are critical for their interactions with β-arrestin-1.

## Discussion

**Figure 5:**
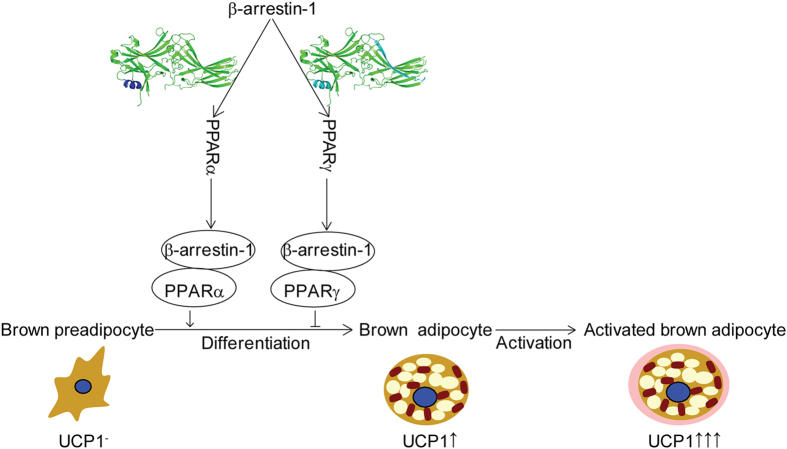
Model of β-arrestin-1 and PPARα/γ in brown fat adipogenesis. Binding of β-arrestin-1 to PPARα and PPARγ dually modulates transcriptional activities of PPARα and PPARγ and regulates brown fat adipogenesis and function.

Function of brown adipose tissue contributes to energy expenditure and adaptive thermogenesis^29^. PPARs, the major regulators in metabolism, play critical regulatory roles in regulating brown fat adipogenesis and function. Activation of PPARα stimulates WAT browning. Meanwhile, PPARγ promotes and maintains the stable differentiation of brown adipocyte in cooperation with diverse cofactors such as C/EBPs and PRDM16^30^. In our study, we found that β-arrestin-1 directly interacts with PPARα and PPARγ. We identified the interaction domain in β-arrestin-1 as well as that in PPARα/γ. Our results show that β-arrestin-1 functions as a cofactor to regulate the activities of PPARα and PPARγ by enhancing PPARα-mediated but repressing PPARγ-mediated transcriptional activity, suggesting a dual regulatory role of β-arrestin-1 for PPARs function in BAT adipogenesis (Fig. 5).

β-arrestins function mainly by binding to diverse partners and play critical roles in regulating various signaling pathways. Studies have shown that dysfunctions of β-arrestins contribute to various disease progressions^31^,^32^. Our previous work showed that *in vivo* expression of β-arrestin-1 repressed adipogenesis in WAT and diet-induced obesity^21^,^22^. Our current study extends the metabolic regulatory function of β-arrestin-1 and provides new evidence that β-arrestin-1 contributes to BAT function in addition to its role in WAT. We showed that genetic ablation of β-arrestin-1 led to an enhanced cold tolerance. Consistent with this result, we observed an induction of thermogenic gene expression, such as *Ucp1* and *Cidea* in BAT of the Arrb1-KO mice. Interestingly, compared with the wild-type littermates, a brown-like phenotype in iWAT was observed in Arrb1-KO mice, which was associated with increased expression of *Ucp1*. However, the functional bi-direction mediation of PPARs activity by β-arrestin-1 need to be confirmed *in vivo*, which is now under investigation.

## Methods

### Animals

Studies were carried out with C57BL/6 mice obtained from Shanghai Laboratory Animal Center, Chinese Academy of Sciences. All mice were maintained in pathogen-free conditions. Animal care and use were in accordance with the guidelines of the Institute of Biochemistry and Cell Biology, Chinese Academy of Sciences. All animal experimental procedures were approved and overseen by the Animal Care and Use Committee of the Shanghai Institute of Biochemistry and Cell Biology, Chinese Academy of Sciences.

### Antibodies and reagents

Mouse anti-β-arrestin-1 polyclonal antibody was from Abmart. Rabbit anti-β-arrestin-1 (A1CT) antibody was a gift from Dr. Robert J. Lefkowitz. Rabbit anti-PPARα polyclonal antibody was purchased from Abcam. Rabbit anti-PPARγ polyclonal antibody was from Santa Cruz Biotechnology. Thrombin protease was obtained from GE Healthcare. ^15^NH_4_Cl was from Cambridge Isotope Laboratories. Inc. Rosiglitazone, GW7647, and n-Dodecyl β-D-maltoside (DDM) were from Sigma.

### Cold exposure studies

Twelve-week-old wild-type and Arrb1-KO mice were housed individually and exposed to 5 °C for 6 hours with free access to water. Core body temperatures were measured with a rectal microprobe thermometer (BAT-12; Physitemp) before and at indicated intervals during cold exposure. After 6 hours of cold exposure, mice were sacrificed and tissues were harvested.

### Immunoprecipitation assay

Endogenous immunoprecipitation experiments were performed as described^27^. Briefly, HepG2 cells were lysated with IP buffer and β-arrestin-1 was immunoprecipitated using mouse anti-β-arrestin-1 antibody overnight, following incubation with protein G affinity gel (Sigma) for 1 h. Immunoprecipitated complexes were eluted and subjected to Western blot. Images were analyzed using the Odyssey infrared imaging system.

### Cloning, Protein expression and purification

Recombinant *rattus* β-arrestin-1 was expressed and purified as reported previously^27^. The ligand binding domains (LBD) of *homo* wild-type PPARα (amino acids 196–468) and PPARγ (amino acids 204–477) and their mutants were cloned into a modified pET28a vector with an N-terminal His6 tag. His6-tagged *homo* RXRα-LBD (amino acids 196–435) was cloned into a pET15b vector. *Homo* PAPRα-LBD, PPARγ-LBD, and RXRα-LBD were expressed in *Escherichia coli* BL21 (DE3)-CodonPlus cells. Protein purifications were performed as described^25^,^33^,^34^. The β-arrestin-1 peptides and the control peptides were commercially synthesized (GL Biochem).

### Pulldown assay

His-tagged PPARα/γ-LBD were mixed with β-arrestin-1 in binding buffer A (20 mM HEPES, pH 8.0, 100 mM NaCl, 1 mM EDTA, 5 mM DTT, and 0.01% DDM) at 25 °C for 30 min. The protein mixtures were then incubated with pre-equilibrated Ni-NTA resins (QIAGEN) in binding buffer B (buffer A with 40 mM imidazole) at 4 °C for 90 min. After washing with binding buffer B, proteins were eluted and analyzed by Western blot.

### Biolayer interferometry assay

Bio-layer interferometry (BLI) is an optical analytical technique for measuring kinetics of interactions in real-time. The biosensor tip surface immobilized with a ligand is incubated with an analyte in solution, resulting in an increase in optical thickness at the biosensor tip and a wavelength shift, which is a direct measure of the change in thickness.

Biolayer interferometry analysis of β-arrestin-1 or RXRα-LBD binding to PPARα-LBD and PPARγ-LBD were studied using Octet Red 96 (ForteBio). 50 μg/ml biotinylated PAPRα-LBD and PPARγ-LBD were immobilized on Streptavidin biosensor (SAs, ForteBio) and the typical immobilization levels were above 3 nm. Ligands-loaded SAs were then incubated with different concentrations of β-arrestin-1 or RXRα-LBD in the kinetics buffer. Global fitting of the binding curves generated a best fit with the 2:1 HL model and the kinetic association and dissociation constants were calculated. The systematic baseline drifts were corrected by subtracting the shifts recorded from sensors loaded with ligands but incubated with no analytes. All binding experiments were performed in solid-black 96-well plates containing 200 μl of solution in each well at 25 °C with an agitation speed of 1000 rpm. Curve fitting, steady state analysis, and calculation of kinetic parameters (k_*on*_, k_*off*_ and K_*D*_) and Rmax parameters were done using Octet software version 7.0 (ForteBio). The goodness of fit for the binding data was assessed by evaluation of the χ^2^ and R^2^ values generated from all the fitting analysis. All the experiments were repeated at least twice.

### Luciferase reporter assay

HEK-293T cells were transiently co-transfected with pGL3-PPRE-Luc and pcDNA3-PPARα/γ. After transfection for 8 hours, cells were treated with the PPARα-specific agonist GW7647 or PPARγ-specific agonist Rosiglitazone for an additional 24 hours before harvesting for luciferase activity measurement using Dual Luciferase Assay System kit (Promega).

### Nuclear magnetic resonance (NMR) spectroscopy

The NMR samples were prepared in a buffer containing 20 mM potassium phosphate, pH 7.4, 100 mM KCl, 2 mM DTT and 10% v/v D_2_O. ^15^N-labeled PPARα-LBD and PPARγ-LBD (0.5 mM) were titrated with β-arrestin-1 peptide up to a molar ratio of 1:10. 2D [^1^H-^15^N]-HSQC spectra were recorded after each addition at 25 °C on Agilent DD2 800-MHz spectrometer equipped with a triple resonance cryo genic probe. Data were processed by NMRPipe and analyzed with NMRView.

### Statistical analysis

The quantitative results are presented as mean ± S.E.M. Unpaired two-tailed Student’s *t* test was used to compare two groups. One-way ANOVA was used to compare three or more groups. Multiple comparisons were analyzed by two-way ANOVA followed by Bonferroni post hoc tests. Differences with *P* value of 0.05 or less were considered statistically significant.

## Additional Information

**How to cite this article**: Wang, C. *et al.* β-arrestin-1 contributes to brown fat function and directly interacts with PPARα and PPARγ. *Sci. Rep.* **6**, 26999; doi: 10.1038/srep26999 (2016).

## Supplementary Material

Supplementary Information

## Figures and Tables

**Table 1:** Kinetics of interaction between β-arrestin-1 and PPARα-LBD.

Ligand	Analyte	Rate constants		K_*D*_ (μM)	Rmax	χ^2^	R^2^
		K_*on*_(M^−1^S^−1^)	K_*off*_(S^−1^)				
PPARα-LBD	β-arrestin-1	1.57 × 10^3^^a^	1.91 × 10^−3^^c^	1.21^e^	0.81^h^	0.03^k^	0.99^l^
		4.86 × 10^4^^b^	8.56 × 10^−2^^d^	1.76^f^	0.40^i^		
				3.60^g^	0.65^j^		
	βarr1M	1.84 × 10^3^^a^	2.81 × 10^−3^^c^	1.53^e^	0.77^h^	0.02^k^	0.99^l^
		1.53 × 10^5^^b^	2.69 × 10^−1^^d^	1.76^f^	0.31^i^		
				3.90^g^	0.51^j^		
	β-arrestin-1 peptide	2.16 × 10^1^^a^	9.07 × 10^−4^^c^	42^e^	0.29^h^	0.05^k^	0.99^l^
		2.66 × 10^3^^b^	1.61 × 10^−1^^d^	60.3^f^	0.11^i^		
				56.03^g^	1.08^j^		
	RXRα-LBD	1.62 × 10^5^^a^	4.63 × 10^−3^^c^	0.029^e^	0.50^h^	0.36^k^	0.97^l^
		7.94 × 10^3^^b^	1.22 × 10^−4^^d^	0.015^f^	0.41^i^		
				0.029^g^	0.10^j^		
PPARα Μ2	β-arrestin-1	2.70 × 10^3^^a^	9.76 × 10^−2^^c^	36.2^e^	0.66^h^	0.05^k^	0.99^l^
		1.45 × 10^3^^b^	2.33 × 10^−3^^d^	1.61^f^	0.43^i^		
				4.10^g^	0.61^j^		

Reported values are representative of a single experiment. Similar results were obtained in replicate experiments.

^a^K_*on*1_,

^b^K_*on*2_: derived from the curve fitting for data with the 2:1 HL model.

^c^K_*off*1_,

^d^K_*off2*_: derived from the curve fitting for data with the 2:1 HL model.

^e^K_*D*1_,

^f^K_*D*2_: derived from the curve fitting for data with the 2:1 HL model.

^g^K_*D*_: obtained from steady state analysis of the secondary plot. Response at equilibrium versus concentration of analyte.

^h^Rmax1,

^i^Rmax2: derived from the curve fitting for data with the 2:1 HL model.

^j^Rmax: obtained from steady state analysis of the secondary plot. Response versus concentration of analyte.

^k^χ^2^: obtained from the curve fitting for data with the 2:1 HL model.

^l^R^2^: obtained from the curve fitting for data with the 2:1 HL model.

**Table 2:** Kinetics of interaction between β-arrestin-1 and PPARγ-LBD.

Ligand	Analyte	Rate constants		K_*D*_ (μM)	Rmax	χ^2^	R^2^
		K_*on*_(M^−1^S^−1^)	K_*off*_(S^−1^)				
PPARγ-LBD	β-arrestin-1	8.21 × 10^4^^a^	2.19 × 10^−1^^c^	2.66^e^	0.76^h^	0.20^k^	0.99^l^
		1.39 × 10^3^^b^	2.93 × 10^−4^^d^	0.21^f^	0.74^i^		
				1.40^g^	0.85^j^		
	βarr1M	2.50 × 10^5^^a^	7.42 × 10^−1^^c^	2.97^e^	0.38^h^	0.14^k^	0.99^l^
		1.30 × 10^3^^b^	1.24 × 10^−3^^d^	0.95^f^	0.25^i^		
				1.70^g^	0.64^j^		
	β-arrestin-1 peptide	5.80^a^	3.23 × 10^−3^^c^	557^e^	0.29^h^	0.04^k^	0.99^l^
		2.53^b^	1.32 × 10^−1^^d^	521^f^	0.16^i^		
				560^g^	0.51^j^		
	RXRα-LBD	1.77 × 10^5^^a^	6.04 × 10^−3^^c^	0.034^e^	1.76^h^	0.90^k^	0.99^l^
		1.82 × 10^4^^b^	8.90 × 10^−6^^d^	0.0005^f^	0.50^i^		
				0.071^g^	2.26^j^		
PPARγ M1	β-arrestin-1	9.15 × 10^4^^a^	8.36 × 10^−1^^c^	9.14^e^	0.68^h^	0.42^k^	0.99^l^
		8.85 × 10^2^^b^	8.16 × 10^−4^^d^	0.92^f^	0.52^i^		
				1.90^g^	0.71^j^		
PPARγ Μ2	β-arrestin-1	1.21 × 10^5^^a^	8.25 × 10^−1^^c^	6.84^e^	0.58^h^	0.02^k^	0.99^l^
		1.05 × 10^3^^b^	5.20 × 10^−3^^d^	4.97^f^	0.25^i^		
				1.50^g^	0.55^j^		

Reported values are representative of a single experiment. Similar results were obtained in replicate experiments.

^a^K_*on*1_,

^b^K_*on2*_: derived from the curve fitting for data with the 2:1 HL model.

^c^K_*off*1_,

^d^K_*off*2_: derived from the curve fitting for data with the 2:1 HL model.

^e^K_*D1*_,

^f^K_*D*2_: derived from the curve fitting for data with the 2:1 HL model.

^g^K_*D*_: obtained from steady state analysis of the secondary plot. Response at equilibrium versus concentration of analyte.

^h^Rmax1,

^i^Rmax2: derived from the curve fitting for data with the 2:1 HL model.

^j^Rmax: obtained from steady state analysis of the secondary plot. Response versus concentration of analyte.

^k^χ^2^: obtained from the curve fitting for data with the 2:1 HL model.

^l^R^2^: obtained from the curve fitting for data with the 2:1 HL model.
