# Supplementary material for: β-arrestin-1 contributes to brown fat function and directly interacts with PPARα and PPARγ
Source: Sci Rep. 2016 Jun 15;6:26999. doi: 10.1038/srep26999 (PMC4908412; doi:10.1038/srep26999)
Supplement: Supplementary Information [file srep26999-s1.pdf]

β-arrestin-1 contributes to brown fat function and directly interacts with PPARα and PPARγ

Congcong Wang[1, 2], Xianglu Zeng[1, 5], Zhaocai Zhou[1], Jian Zhao[1, 4, *], Gang Pei[1, 3, *]

[1] *State Key Laboratory of Cell Biology, Institute of Biochemistry and Cell Biology, Shanghai Institutes for Biological Sciences, Chinese Academy of Sciences, 320 Yueyang Road, Shanghai 200031, China*

[2] *Graduate School, University of Chinese Academy of Sciences, Chinese Academy of Sciences, 320 Yueyang Road, Shanghai 200031, China*

[3] *School of Life Science and Technology, Tongji University, Shanghai 200092, China*

[4] *Translational Medical Center for Stem Cell Therapy, Shanghai East Hospital, School of Medicine, Tongji University, Shanghai 200120, China*

[5] *Shanghai Key Laboratory of Signaling and Disease Research, Laboratory of Receptor-based Bio-medicine, School of Life Sciences and Technology, Tongji University, Shanghai 200092, China*

** Correspondence and requests for materials should be addressed to Gang Pei (email: gpei@sibs.ac.cn) and Jian Zhao (email: jzhao@sibcb.ac.cn).*

Supplementary Information

Supplementary Figures

Figure S1 Targeted disruption of *Arrb1* gene in Mice.

Figure S2 Body weights and blood glucose levels of the Arrb1-KO mice and their wild-type littermates.

Figure S3 Kinetic analysis for different immobilization levels of PPARα, PPARγ and RXRα.

Figure S4 Comparison of the fitting models on the binding curves of PPARα/γ-LBD with β-arrestin-1 or RXRα-LBD.

Figure S5 The kinetics of β-arrestin-1 binding to PPARα-LBD and PPARγ-LBD in the presence of RXRα-LBD.

Figure S6 The kinetics of Gαs binding to β-arrestin-1 and βarr1M.

Figure S7 Effects of β-arrestin-1 peptide on the interactions between RXRα-LBD and PPARα/γ-LBD.

Figure S8 The kinetics of PPARα/γ-LBD binding to β-arrestin-1 peptide mutants.

Figure S9 The kinetics of βarr1M binding to PPARγ M1 and M2.

Figure S10 The residual plots for the fitting curves in Fig. 3 and Fig. 4.

Figure S11 Full-length blots for Fig. 2 and Fig. 3.

Supplementary Table

Table S1 Primer List for RT-PCR.

Table S2 Molecular Weight (MW) of the indicated proteins and peptides.

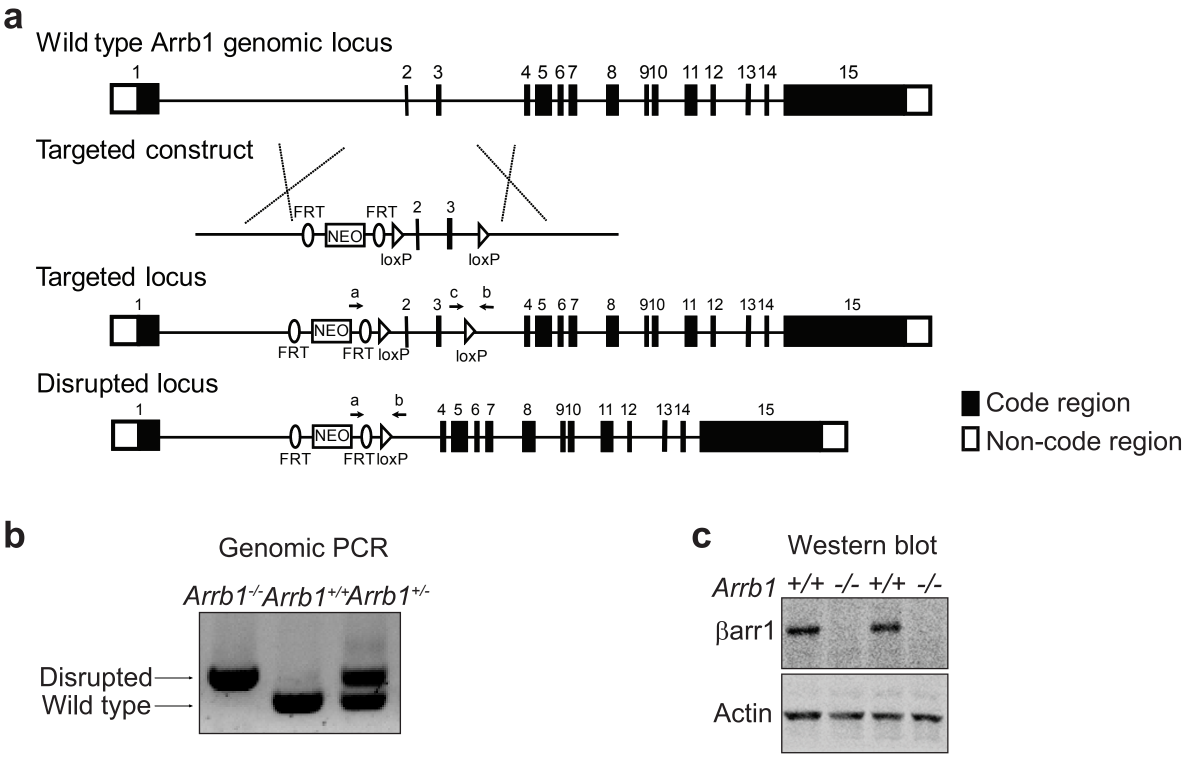

Figure S1. Targeted disruption of *Arrb1* gene in Mice. (**a**) Schematic of *Arrb1* gene-targeting strategy. Excision of the sequences between the loxP sites by the *Cre* recombinase deletes 2-3 exons in *Arrb1*. The locations of the primers a, b, and c used for genomic PCR analysis are indicated. C Representative PCR analysis of genomic DNA from the homozygous , wild-type and heterozygous mice. The migrated position of the fragments derived from the wild-type and disrupted alleles are indicated. (**c**) Western blot analysis of the expression of β-arrestin-1 in BAT of the wild-type and Arrb1-KO mice.

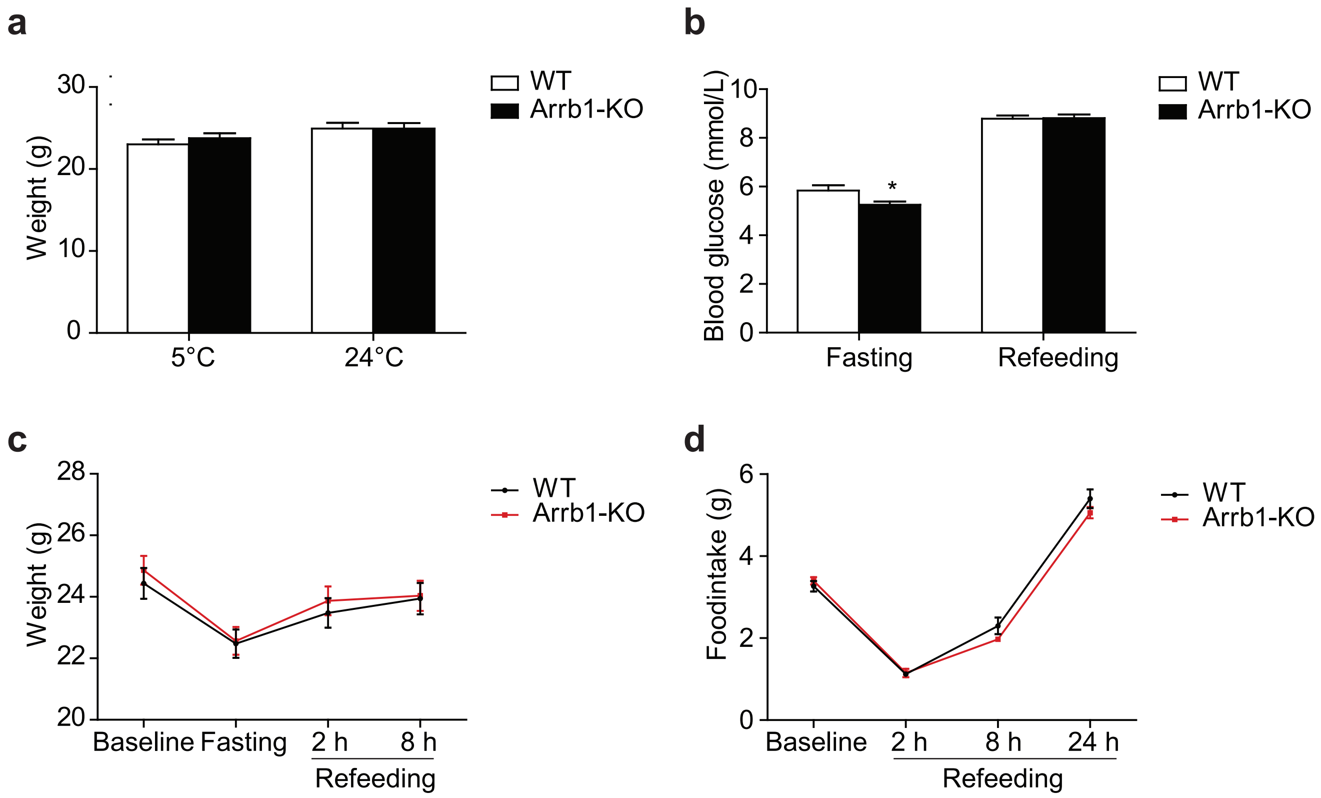

Figure S2. Body weights and blood glucose levels of the Arrb1-KO mice and their wild-type littermates. (**a**) Body weights of the wild-type (n=13) and Arrb1-KO (n=13) mice for each group exposed to either 5°C or 24°C. Data are shown as means ± S.E.M. (**b**) Blood glucose levels of the wild-type (n=25) and Arrb1-KO (n=25) mice after fasting and refeeding. Mice were fasted for 16 h (from 17:00 to 9:00) and then refed with a regular chow diet for 2 h (9:00-11:00). Data are presented as means ± S.E.M. *, $p < 0.05$ versus the wild-type group. (**c**) Body weights of the wild-type (n=20) and Arrb1-KO (n=20) mice after fasting for 16 h and refeeding for 2 h or 8 h. Mice were fasted for 16 h (from 17:00 to 9:00) and then refed with a regular chow diet for 8 h (9:00-17:00). Data are presented as means ± S.E.M. (**d**) Food intakes of the wild-type (n=14) and Arrb1-KO (n=14) mice after refeeding for 2 h, 8 h, and 24 h. Mice were fasted for 16 h (from 17:00 to 9:00) and then refed with a regular chow diet for 24 h. Data are presented as means ± S.E.M.

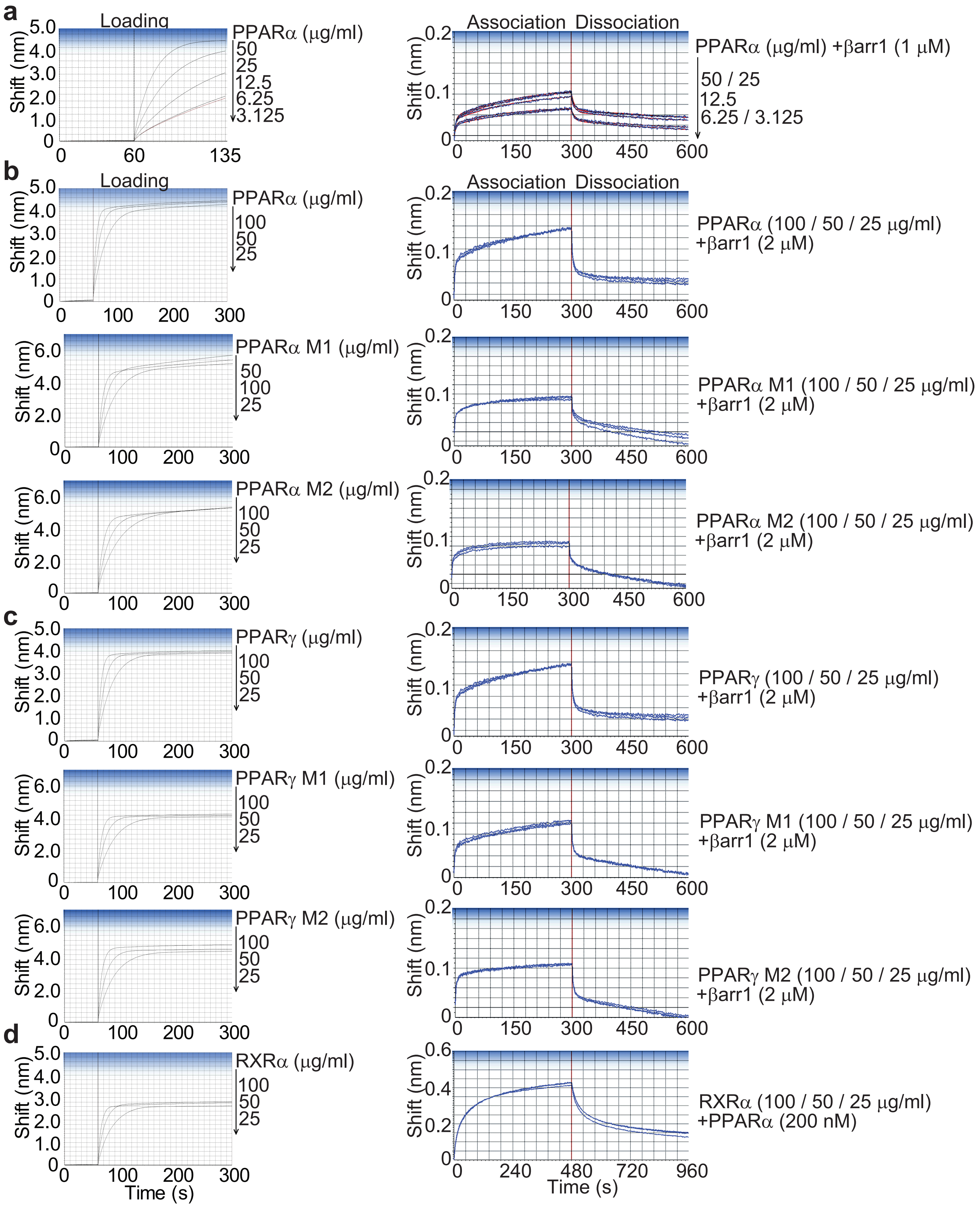

Figure S3. Kinetic analysis for different immobilization levels of PPARα, PPARγ and RXRα. **(a)** Biolayer interferometry analysis for the interaction of PPARα with β-arrestin-1. Streptavidin biosensors (SAs) were immobilized with 50-3.125 μg/ml biotinylated PPARα-LBD for 70 s. The SAs were then incubated with 1 μM β-arrestin-1 at 25°C. **(b, c)** Kinetic analysis for the interaction of PPARα/γ with β-arrestin-1. Purified 100-25 μg/ml biotinylated wild-type PPARα/γ-LBD, PPARα/γ M1 and M2 were loaded onto the SAs for 240 s, and then incubated with 2 μM β-arrestin-1 at 25°C. **(d)** Kinetic analysis for the interaction of RXRα-LBD with PPARα-LBD. Purified 100-25 μg/ml biotinylated RXRα-LBD were immobilized onto the SAs for 240 s and incubated with 200 nM PPARα at 25°C. All the association/dissociation curves were shown on the right.

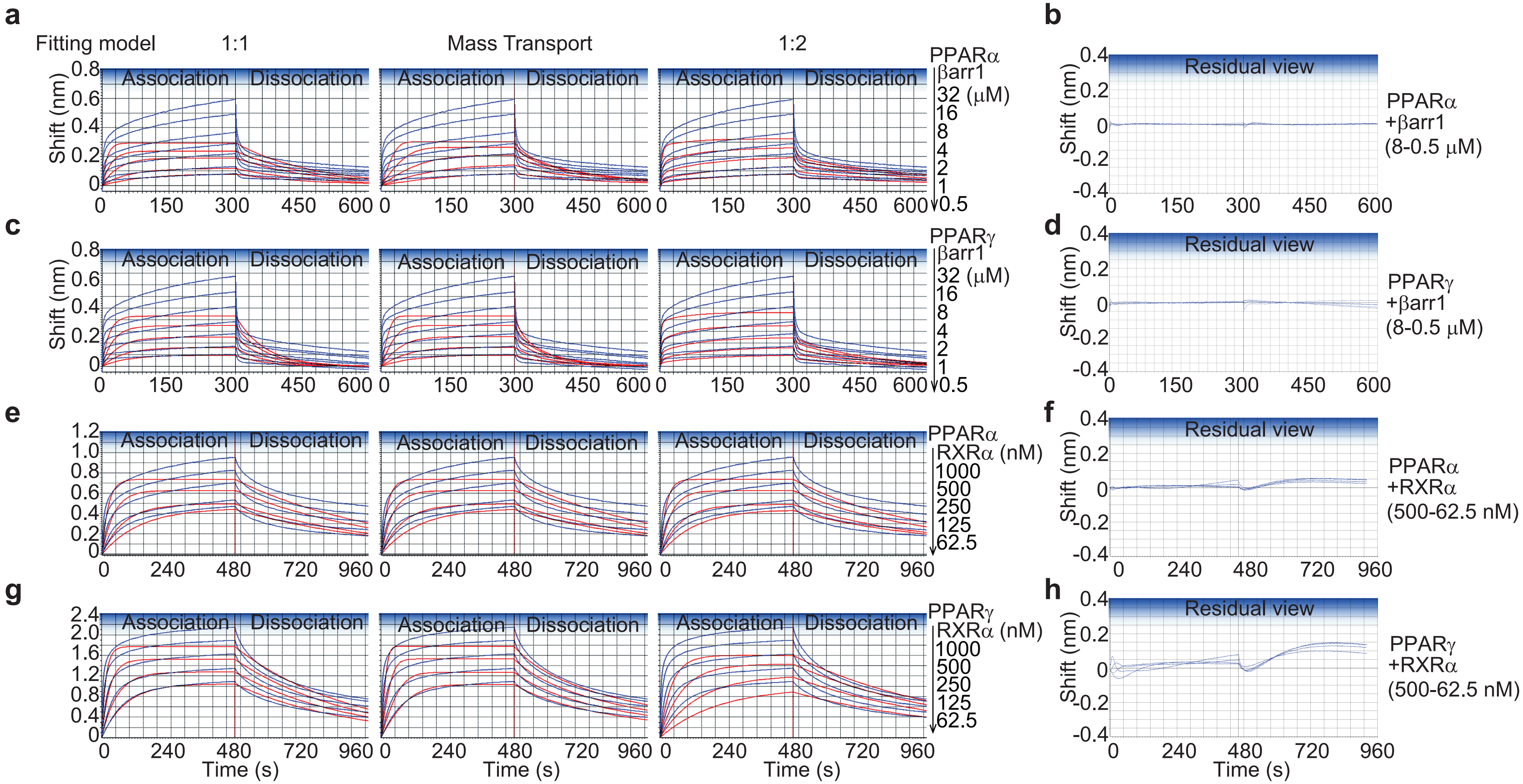

Figure S4. Comparison of the fitting models on the binding curves of PPARα/γ-LBD with β-arrestin-1 or RXRα-LBD. (**a-d**) The binding curves of PPARα-LBD or PPAR**γ**-LBD with β-arrestin-1 were fitted using the 1:1, Mass Transport, and 1:2 BA models. The residual plots generated from a 2:1 HL model were shown on the right. (**e-h**) The binding curves of PPARα-LBD or PPAR**γ**-LBD with RXRα-LBD were fitted using the 1:1, Mass Transport, and 1:2 BA models. The residual plots generated from a 2:1 HL model were shown on the right. The experimental data are represented by blue lines and the curve fitting data are indicated by red lines.

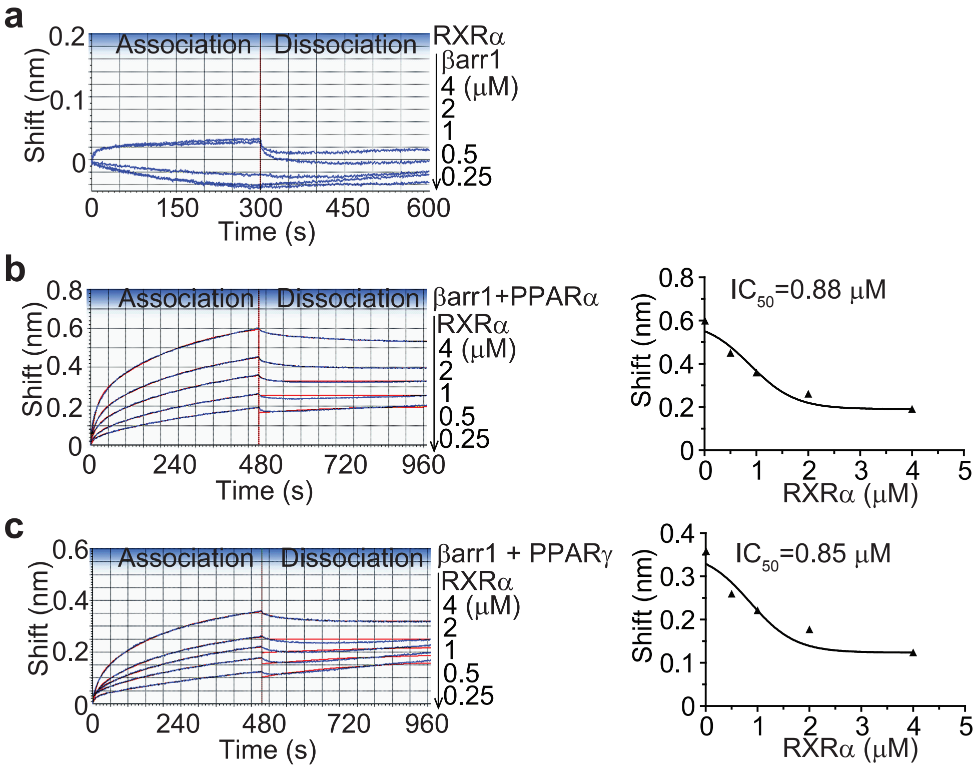

Figure S5. The kinetics of β-arrestin-1 binding to PPARα-LBD and PPARγ-LBD in the presence of RXRα-LBD. (**a**) Biolayer interferometry analysis of the interaction between β-arrestin-1 and RXRα-LBD. Streptavidin biosensor (SA) immobilized with 50 μg/ml biotinylated RXRα-LBD was incubated in wells containing different concentrations of purified β-arrestin-1 at 25°C. (**b, c**) The kinetics of β-arrestin-1 binding to PPARα-LBD and PPARγ-LBD in the presence of RXRα-LBD. Purified 50 μg/ml β-arrestin-1 was loaded on SAs and incubated with 200 nM PPARα-LBD and PPARγ-LBD in the presence of a serial dilution of RXRα-LBD at 25°C. The IC_{50} values were estimated from the plots of maximum responses versus concentrations.

a

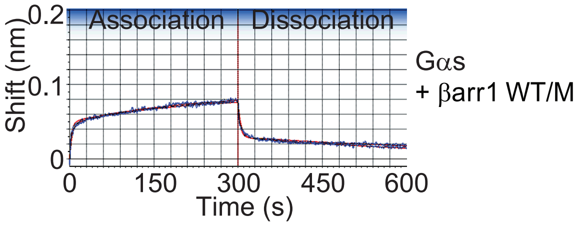

Figure S6. The kinetics of Gαs binding to β-arrestin-1 and βarr1M. (**a**) Biolayer interferometry analysis of the interactions between Gαs and β-arrestin-1 or βarr1M. Streptavidin biosensors immobilized with 50 μg/ml biotinylated Gαs were incubated with 2 μM β-arrestin-1 or βarr1M at 25°C.

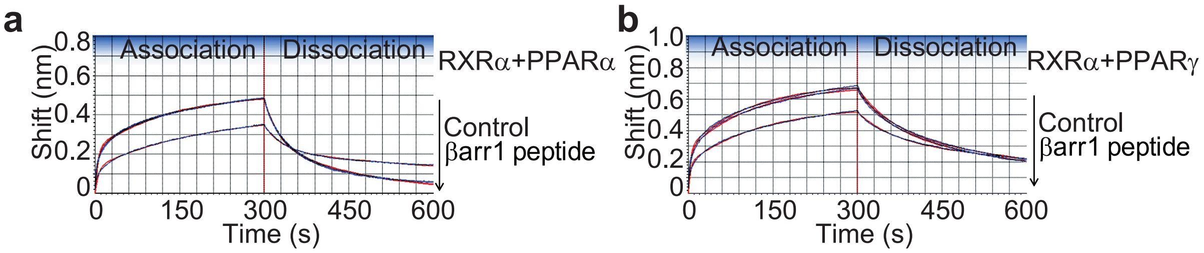

Figure S7. Effects of β-arrestin-1 peptide on the interactions between RXRα-LBD and PPARα/γ-LBD. (**a, b**) The kinetics of RXRα-LBD binding to PPARα-LBD and PPARγ-LBD in the presence of β-arrestin-1 peptide. 50 μg/ml biotinylated RXRα-LBD was loaded on SAs and incubated with 200 nM PPARα-LBD or PPARγ-LBD in the presence of 0.4 mM β-arrestin-1 peptide or an irrelevant peptide at 25°C.

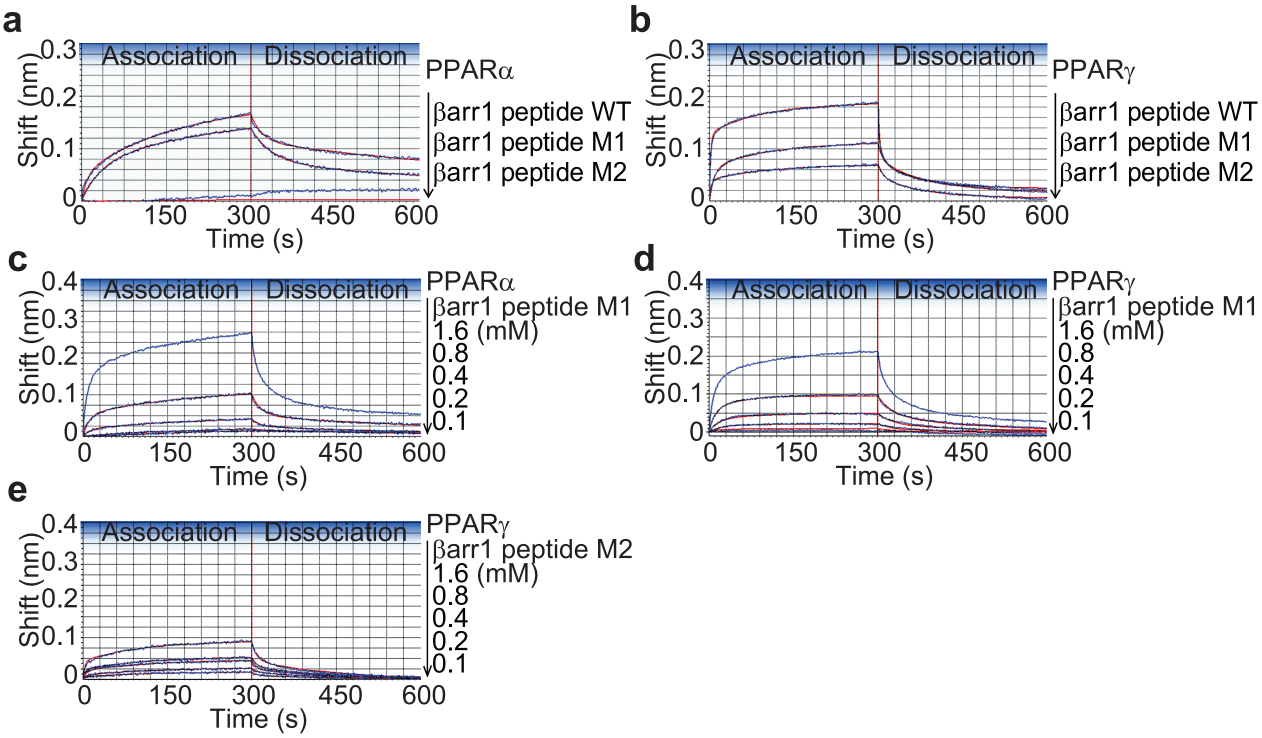

Figure S8. The kinetics of PPARα/γ-LBD binding to β-arrestin-1 peptide mutants. (**a, b**) Biolayer interferometry analysis of the interactions between PPARα/γ-LBD and β-arrestin-1 peptide M1 or M2. 50 μg/ml PPARα/γ-LBD-loaded SAs were incubated with 0.8 mM β-arrestin-1 peptide M1 or M2 at 25°C. (**c, d, e**) The kinetic analysis of PPARα/γ-LBD binding to β-arrestin-1 peptide M1 or M2. Purified 50 μg/ml PPARα/γ-LBD were loaded on SAs and incubated with different concentrations of β-arrestin-1 peptide M1 or M2 at 25°C. The experimental data are represented by blue lines and the curve fitting data are indicated by red lines.

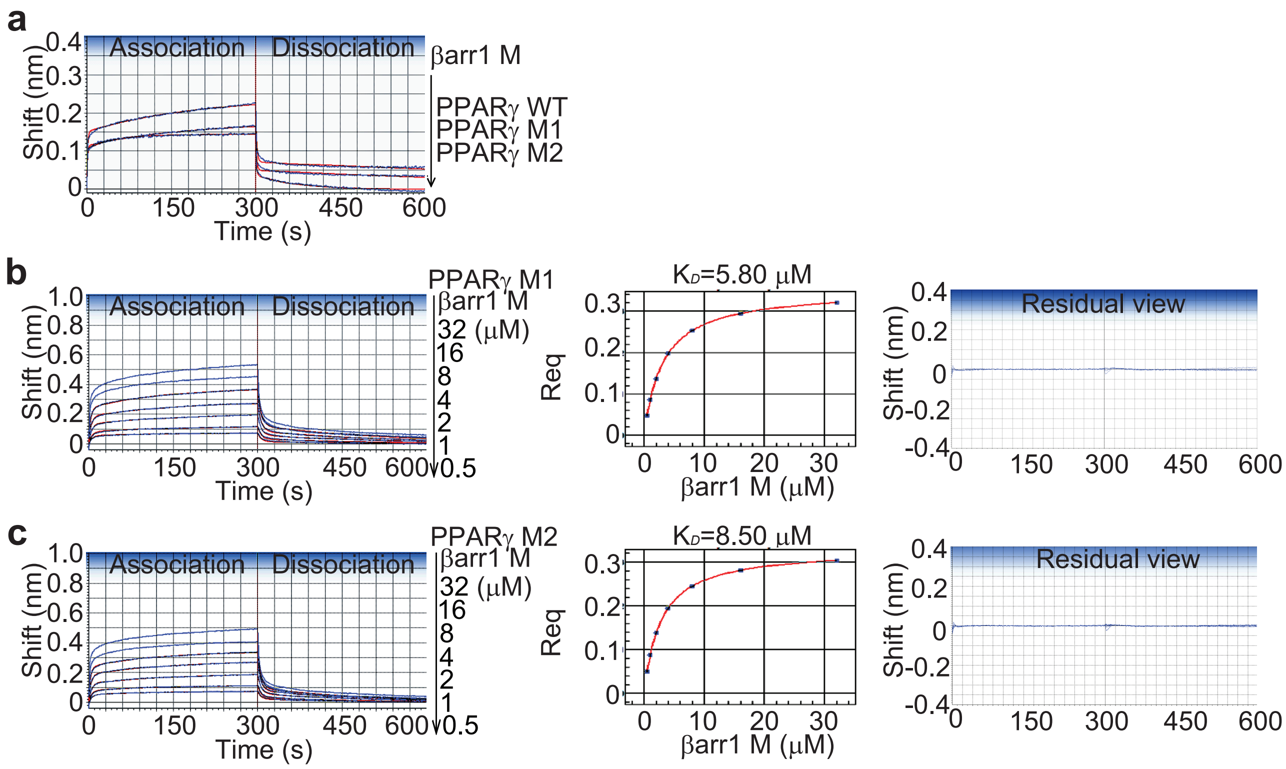

Figure S9. The kinetics of βarr1M binding to PPARγ M1 and PPARγ M2.
(**a**) Interactions between βarr1M and PPARγ-LBD L311G/N312G (PPARγ M1) or D380A (PPARγ M2) using the ForteBio Octet Red instrument. Purified 50 μg/ml wild-type PPARγ-LBD, PPARγ M1, and PPARγ M2 were immobilized on SAs and incubated with 2 μM βarr1M at 25°C. (**b, c**) The kinetic analysis of βarr1M binding to PPARγ M1 and PPARγ M2, respectively. The SAs immobilized with 50 μg/ml PPARγ M1 and PPARγ M2 were incubated with different concentrations of purified βarr1M at 25°C. The steady state analysis and K_D of the binding curves were shown in the middle. The 2:1 HL model was used to fit the binding curves and the residual plots were shown on the right. The experimental data are represented by blue lines and the curve fitting data are indicated by red lines.

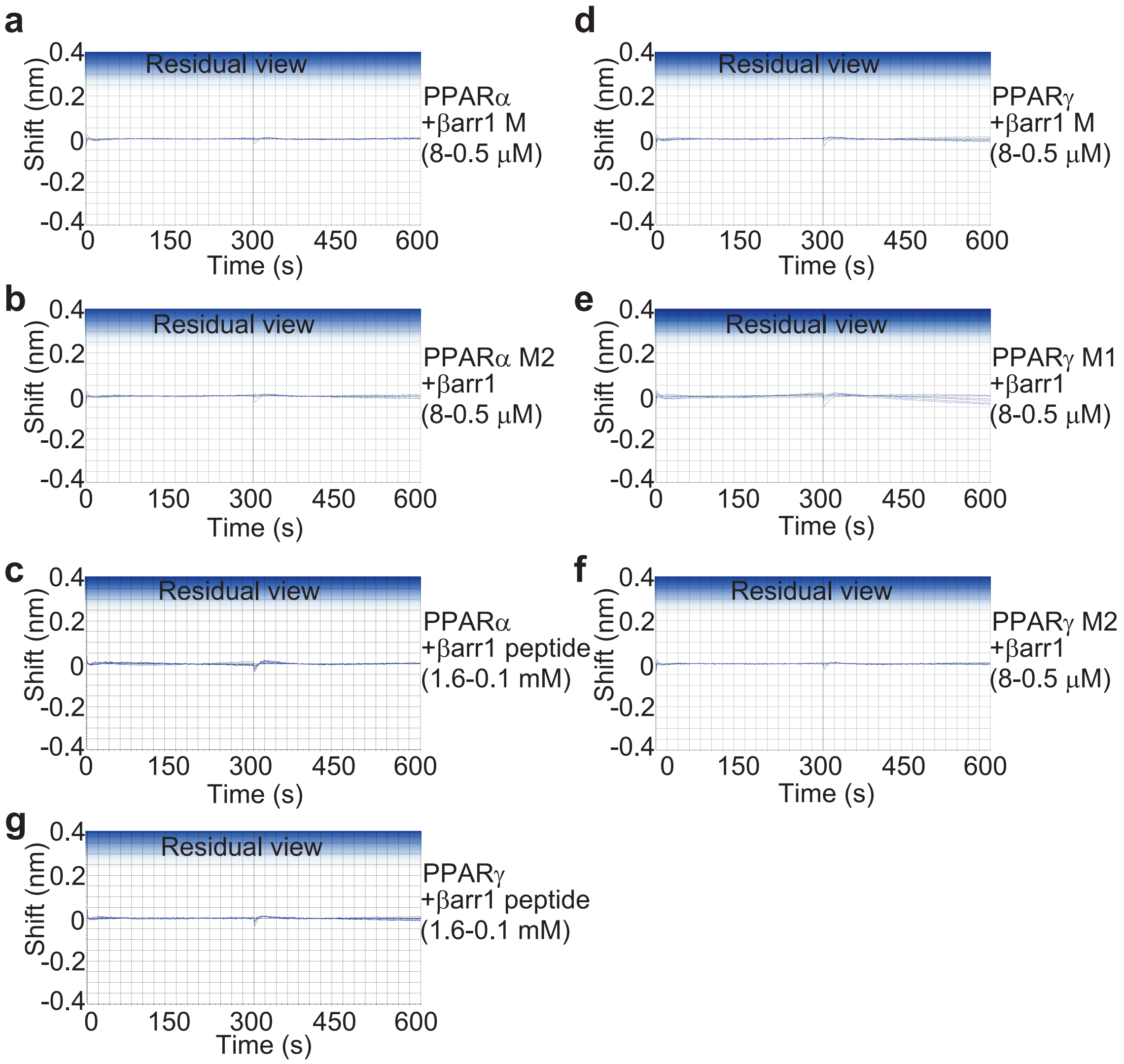

Figure S10. The residual plots from the fitting curves in Fig. 3 and Fig. 4. (**a, b, c**) The residual plots from the fitting curves of wild-type PPARα-LBD or PPARα M2 binding to βarr1 and βarr1 peptide. (**d-g**) The residual plots from the fitting curves of wild-type PPARγ-LBD, PPARγ M1 or PPARγ M2 binding to βarr1 and βarr1 peptide. The 2:1 HL model was used to fit all the binding curves.

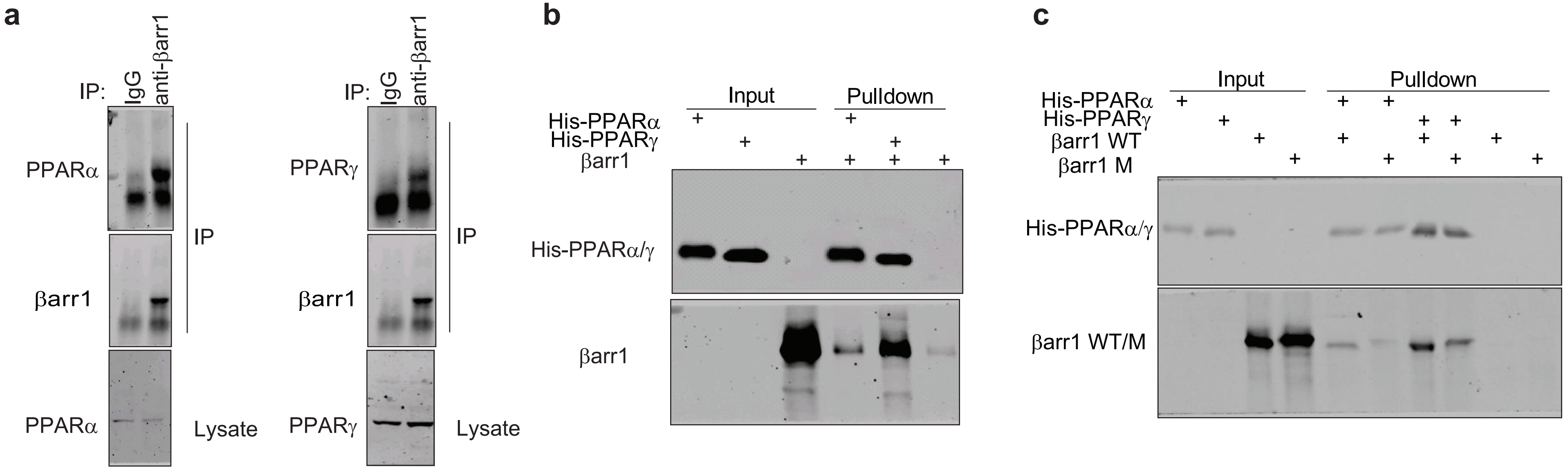

Figure S11. Full-length blots for Fig. 2 and Fig. 3. (**a**) Full-length blots for Fig. 2a. (**b**) Full-length blots for Fig. 2b. (**c**) Full-length blots for Fig. 3b.

Supplementary Table

Table S1. Primer List for RT-PCR

RT-PCR	Forward 5'→3'	Reverse 5'→3'
Ucp1	CACCTTCCCGCTGGACACT	CCCTAGGACACCTTTATACCTAATGG
PGC1α	GCGCCGTGTGATTTACGTT	AAAACTTCAAAGCGGTCTCTCAA
PGC1β	TCCTGTAAAAGCCCGGAGTAT	GCTCTGGTAGGGGCAGTGA
Cox7a	CAGCGTCATGGTCAGTCTGT	AGAAAACCGTGTGGCAGAGA
CPT1β	CTGTTAGGCCTCAACACCGAAC	CTGTCATGGCTAGGCTGTACAT
Cidea	ATCACAACTGGCCTGGTTACG	TACTACCCGGTGTCCATTTCT
PPARα	TCAGGGTACCACTACGGAGT	CTTGGCATTCTTCCAAAGCG
PPARγ	GCATGGTGCCTTCGCTGA	TGGCATCTCTGTGTCAACCATG
Prdm16	CAGCACGGTGAAGCCATTC	GCGTGCATCCGCTTGTG

Table S2. Molecular Weight (MW) of the indicated proteins and peptide

**Proteins and peptides**	**MW (Dalton)**
PPARα-LBD	30,865
PPARα M1	30,751
PPARα M2	30,821
PPARγ-LBD	31,283
PPARγ M1	31,170
PPARγ M2	31,239
β-arrestin-1	47,019
βarr1M	46,935
RXRα-LBD	26,823
β-arrestin-1 peptide	2,431
β-arrestin-1 peptide M1	2,263
β-arrestin-1 peptide M2	2,495
The irrelevant peptide	2,688
